# Psychometric testing of the 10-item perceived stress scale for Chinese nurses

**DOI:** 10.1186/s12912-023-01602-4

**Published:** 2023-11-15

**Authors:** Xiaoyu Du, Xiqin Liu, Yajun Zhao, Song Wang

**Affiliations:** 1 Department of Radiology and Huaxi MR Research Center (HMRRC), Functional and Molecular Imaging Key Laboratory of Sichuan Province, West China Hospital, Sichuan University, Chengdu, China; 2 School of Education and Psychology, Southwest Minzu University, Chengdu, China

**Keywords:** Perceived Stress Scale, Chinese nurses, Stress, Reliability, Validity

## Abstract

**Background:**

Nurses bear a lot of stressors at work. The 10-item Perceived Stress Scale (PSS-10) is a widely used self-reported scale for measuring the global perception of stress. However, there is a lack of use of the PSS-10 in Chinese nurses. This study aimed to test the psychometric properties of the PSS-10 among Chinese nurses.

**Methods:**

A total of 708 Chinese nurses completed the PSS-10, the Big Five Inventory (BFI), and the Depression Anxiety and Stress Scale (DASS). Confirmatory factor analysis (CFA) tested the factor structure of the PSS-10. Cronbach’s α and test-retest correlation examined the scale reliability. Pearson correlation and hierarchical regression analyses tested the convergent, discriminant and criterion validity of the PSS-10.

**Results:**

CFA revealed that a two-factor model fits the structure of the PSS-10 in Chinese nurses (*χ*^2^/*df =* 6.25, *p* < 0.001; comparative fit index [CFI] = 0.94, non-normal fit index [NNFI] = 0.92, Tucker-Lewis index [TLI] = 0.91, root mean square error of approximation [RMSEA] = 0.08, standardized root mean square residual [SRMR] = 0.05). The scale demonstrated adequate internal consistency (α = 0.86) and test-retest reliability (*r* = 0.66, *p <* 0.001), satisfactory convergent and discriminant validity with relations to Big Five personalities, as well as good criterion validity such that the PSS-10 score could explain incremental variance in predicting anxiety, depression and stress.

**Conclusions:**

Our findings suggest that PSS-10 is a reliable and valid measure of perceived stress among Chinese nurses and can be used in future research and practice on stress management and coping in Chinese nurses.

**Supplementary Information:**

The online version contains supplementary material available at 10.1186/s12912-023-01602-4.

## Background

Stress is significantly associated with many psychological health conditions such as depression, anxiety, and fatigue [1–3]. Stress in the working environment has proved to have a close relation with decreased job satisfaction and increased burnout of healthcare workers, which may further lead to poor job performance [4]. Nursing has been confirmed as an occupation with high levels of stress. For example, a study of a sample of trauma nurses in New York has shown that the nurses’ perceived stress scores are higher than the average perceived stress scores observed in previous studies focusing on other populations [5]. An epidemiological study of 850 Chinese nurses revealed a prevalence of depression, anxiety and stress at 35.8%, 37.3% and 41.1% respectively, which are almost three times higher than in a survey with a cohort of 5719 Chinese general adults [6]. Moreover, a study concerning occupational stress in Iranian nurses found that 78.4% of respondents reported high work pressure [7]. Long working hours, irregular schedules, heavy workloads, and lack of positive professional recognition are all contributors to stress perception among nurses [7, 8]. Therefore, it is of great necessity to pay more attention to stress and strengthen stress management and prevention programs (e.g., mindfulness-based intervention [9]) for nurses.

Perceived stress refers to the extent to which situations in one’s life are evaluated as stressful, uncontrollable and unpredictable [10]. In contrast to the research focusing on the type or frequency of stressful events, perceived stress reflects an individual’s global subjective perception of stress and his/her ability to deal with it [11]. As a self-reported instrument for measuring perceived stress, the Perceived Stress Scale (PSS) was initially developed as the 14-item PSS, which was later simplified into the 10-item PSS (PSS-10) and 4-item PSS (PSS-4) for phone screenings [12]. A review of PSS has shown that the psychometric properties of the PSS-10 are superior to those of the PSS-14 and PSS-4, and thus recommending the use of PSS-10 for measuring perceived stress both in practice and research [13]. This scale has two dimensions: perceived helplessness (with loadings on six negative items: 1, 2, 3, 6, 9, and 10) and perceived self-efficacy (with loadings on four positive items: 4, 5, 7, and 8) [12, 13]. The PSS-10 has been widely used in investigating stressful life events, physical and mental illness, and stress management and prevention [14, 15]; and it has been translated into over 25 languages and employed in more than 20 countries [16]. The PSS-10 demonstrates internal consistency with Cronbach’s α ranging from 0.80 to 0.86 in different areas of research in various countries, such as Swedish adults [17], Vietnamese older women [16], and Iranian infertile women [18]. It also shows criterion validity with significant relations to distress symptoms including anxiety (*r* = 0.43 to 0.67, *p* < 0.001) and depression (*r* = 0.42 to 0.62, *p* < 0.001) [19, 20].

Although the PSS-10 has been validated in many countries as an economical and effective stress assessment tool, there is a lack of use of the PSS-10 in Chinese nurses. Leung et al. first compared the appropriateness of the three versions of PSS in 1860 cardiac patients with poor smoking habits in Hong Kong, and they found that the PSS-10 had higher internal consistency (α = 0.83) and was suitable for the promotion program [21]. Further psychometric evidence of PSS-10 has been evaluated in different Chinese populations (e.g., people with common mental disorders [22], patients with systemic lupus erythematosus [23], elderly service workers [24], policewomen [25], and general adolescents [20]). The findings of these studies revealed adequate internal consistency (α > 0.7), and satisfied criterion validity with significant links to anxiety (*r* = 0.29 to 0.68, *p* < 0.001) and depression (*r* = 0.43 to 0.67, *p* < 0.001) [20, 22–25]. However, to our knowledge, the psychometric properties of PSS-10 in Chinese nurses remain unexamined. The availability of human resources for health is extremely insufficient in China. Up to 2019, the density of nurses and midwives in China is 31.6 per 10,000 people, which is far lower than the requirement of an 80% universal health coverage target (70.6 per 10,000 population) [26]. More than a quarter of nurses suffer from burnout, depression or anxiety in China; and long working hours (more than 55 h per week) and frequent night shifts (monthly > 4 times) are held accountable for this stressor [27]. Given that perceived stress is an important risk factor for physical and psychological health problems among nurses [28], a valid and reliable stress measurement is particularly necessary to understand the perceived stress levels of Chinese nurses.

This study aimed to examine the psychometric properties of PSS-10 in a sample of Chinese nurses. First, confirmatory factor analysis (CFA) was conducted to evaluate the factor structure of PSS-10. Second, Cronbach’s α and test-retest correlation analyses were calculated to evaluate the reliability of PSS-10. Third, we examined the convergent validity and discriminant validity of PSS-10 by correlating scores between PSS-10 and Big Five personalities. Particularly, general personality traits have been identified as playing an important role in perceiving stressors and assigning meaning to them [29]. The five-factor model is the most widely accepted model of general personalities, where the five factors are neuroticism, extraversion, agreeableness, conscientiousness, and openness [30]. Evidence has indicated that individuals high on neuroticism are more likely to suffer high levels of stress, anxiety, depression, tension, sadness, and nervousness [31]. A recent meta-analytic review has revealed that all of the Big Five personalities are significantly linked with perceived stress, and the absolute effect size of the neuroticism-perceived stress link (r = 0.36) is larger than that of the links of other Big Five personalities with perceived stress (r = -0.14 to -0.07) [32]. Finally, we measured the criterion validity by testing whether PSS-10 scores could predict anxiety, depression and stress beyond other sociodemographic variables using hierarchical regression analyses. Specifically, we used standard anxiety and depression scales as the measure of predictive criterion, given that perceived stress has been found to be a stable predictor for depression and anxiety in different populations [33–38]. In addition, we used a well-validated stress scale as the measure of concurrent criterion.

## Methods

### Study type

This is a cross-sectional survey study aimed at examining the psychometric properties of the PSS-10 in Chinese nurses. The present study was conducted in line with the COnsensus-based Standards for the selection of health status Measurement INstruments (COSMIN) guidelines for scale validation [39] (the details: see **Supplemental Material**).

### Sample and sampling

A total of 756 nurses participated in this survey as part of a larger project aimed at examining the determinants of mental health and job performance among nurses in hospitals in southwest China [40]. Six hospitals in Chengdu and Kunming were selected using convenience sampling. Participants were included based on the following criteria: (1) having no history of neurological or psychiatric illnesses; (2) working at least one year in clinical nursing or nursing management; and (3) obtaining a nurse qualification certificate and registering as a nurse in China. Participants were excluded if they were: (1) retired; (2) training or practice nurses; and (3) on leave during the testing period. Of all participants, there were 708 valid responses and 48 participants who failed to pass the bogus items were excluded (see **Data collection**). Table 1 lists the sociodemographic characteristics of the valid participants. Besides, 182 participants from one of the included hospitals completed the retest of PSS-10 after three months.

**Table 1:** Sociodemographic characteristics of the sample (*N* = 708)

Variable	Mean ± SD (Range) N%
Sex	
Female	642 (90.7%)
Male	66 (9.3%)
Age (years)	31.74 ± 7.39 (18–55)
Educational level	
Graduate degree	4 (0.6%)
Bachelor degree	397 (56.1%)
College degree	270 (38.1%)
Secondary vocational degree	37 (5.2%)
Marital status	
Married	482 (68.1%)
Unmarried	198 (27.9%)
Divorced	26 (3.7%)
Widowed	2 (0.3%)
Professional title	
Vice senior	10 (1.4%)
Intermediate	170 (24.0%)
Primary	365 (51.6%)
None	163 (23.0%)
Length of nursing work (years)	10.67 ± 8.13 (1–39)

*Note.* N: number; SD: standard deviation

### Research measures

The research measures included demographic information surveys, the PSS-10, the 44-item Big Five Inventory (BFI-44), and the 21-item Depression Anxiety and Stress Scale (DASS-21). All measures were written in the subjects’ native language (i.e., Mandarin Chinese). The demographic information surveys included sex, age, education level, marital status, professional title, and length of nursing work.

***PSS-10.*** This is a self-reported scale that assesses how often each item occurred in the past month for the participants [12]. Scores on each item are rated on a 5-point Likert scale ranging from 1 (never) to 5 (always). The ten items included six negative items (1, 2, 3, 6, 9, and 10), interpreted as perceived helplessness, and four positive items (4, 5, 7, and 8), interpreted as perceived self-efficacy [12, 13]. The scores for positive items need to be inverted to calculate the total score and higher scores indicate higher levels of perceived stress. The Chinese version of PSS-10 used in this study was revised by Chu & Kao, which has shown satisfactory reliability and validity in different Chinese samples [20, 41, 42].

***BFI-44.*** The Big Five personality model proposes that individual personality consists of five basic dimensions: extraversion, neuroticism, conscientiousness, agreeableness, and openness [43]. As a popular measure of Big Five personalities, the BFI-44 includes 44 items that are rated on a 5-point Likert scale ranging from 1 (disagree strongly) to 5 (agree strongly). Prior studies have shown adequate validity and reliability of the BFI-44 in different Chinese samples [44–46]. The Cronbach’s αs for BFI-44 subscales in this study were acceptable: extraversion (0.72), agreeableness (0.72), conscientiousness (0.80), neuroticism (0.78), and openness (0.78).

***DASS-21.*** This is a set of three self-report scales designed to measure the emotional states of anxiety, depression, and stress experienced in the past week [47]. Each scale includes 7 items, and participants are asked to respond to each item from 1 (not at all) to 4 (very much so). The DASS-21 has shown satisfactory psychometric properties in different Chinese samples [48, 49]. The Cronbach’s αs for anxiety, depression and stress of DASS-21 in this study were 0.85, 0.86, and 0.86, respectively, indicating adequate internal reliability.

### Data collection

All participants were recruited via a notification to introduce the study from the nursing department of the hospitals. They completed a multi-section questionnaire survey via Sojump (http://www.sojump.com), an efficient, reliable and valid online data collection website, which can avoid missing questions [50]. Each nurse completed the survey anonymously (i.e., no name information was collected) under the guidance of an investigator at each hospital who had been trained by the researchers. The investigator needed to answer questions from participants according to unified guidelines. To ensure that participants filled in the questionnaires honestly and discriminately, we used bogus items (e.g., I have five fingers on my left hand) that had only one correct answer in the tests [51] and 48 participants were excluded during this process. This study was approved by the local research ethics committee of West China Hospital and online informed consent was obtained from all participants before the investigation.

### Data analyses

First, we calculated descriptive statistics for each item of PSS-10 using SPSS 26.0 (IBM, New York, NY, USA), including means, standard deviations (SD), skewness, kurtosis, and discrimination index (DI). Skewness is a measure of asymmetry and kurtosis is a measure of the peakedness of the distribution [52], and the absolute values of skewness and kurtosis smaller than 1 represent a normal distribution for the scores [53]. DI is an indicator to determine the extent to which each item distinguishes individuals with high scores from those with low scores [54, 55]. In general, DI should not be lower than 0.20, otherwise, the item would be considered too easy or too difficult; an item with DI greater than 0.40 is considered excellently acceptable [56].

Second, given that previous studies have established a two-factor structure model of the PSS-10 [17, 20, 57], a CFA was performed with AMOS 26.0 (IBM, New York, NY, USA) to test the two-factor model in the current sample. We calculated the comparative fit index (CFI), non-normal fit index (NNFI), Tucker-Lewis index (TLI), root mean square error of approximation (RMSEA), and standardized root means square residual (SRMR) to evaluate the model’s fitness; and the results with CFI > 0.90, NNFI > 0.90, TLI > 0.90 and RMSEA < 0.08, SRMR < 0.08 can be considered a good fit [58, 59].

Third, Cronbach’s αs were calculated to evaluate the scale’s internal reliability and test-retest correlation analyses were performed to assess the test-retest reliability of the PSS-10. Then, Pearson correlation coefficients between scores of the PSS-10 and the BFI-44 were computed to evaluate the convergent and discriminant validity of the PSS-10. Next, we evaluated the criterion validity of the PSS-10 by testing whether the PSS-10 scores explained additional variance in predicting anxiety, depression and stress based on hierarchical regression analyses. In the hierarchical regression model, the first-level variables were sex, age, education level, professional title, length of nursing work, and Big Five personalities, while PSS-10 scores were treated as a second-level predictor variable, with the dependent variables being anxiety, depression and stress, respectively.

## Results

### Item descriptive statistics

Descriptive statistics for the PSS-10 items are presented in Table 2. The means for each item ranged from 2.44 to 3.16 and SD ranged from 0.73 to 0.94. The absolute skewness and kurtosis values were smaller than 1, indicating that the scores of the items were normally distributed [53]. The DI of each item was greater than 0.20, ranging from 0.30 to 0.44, implying that each item of PSS-10 was well differentiated.

**Table 2:** Descriptive statistics of PSS-10 items

Item	Mean	SD	Skewness	Kurtosis	DI
1. Been upset	2.86	0.88	0.20	0.20	0.41
2. Unable to control	2.50	0.94	0.44	0.11	0.41
3. Stressed	2.69	0.94	0.40	0.05	0.41
4. Felt confident	2.62	0.88	0.17	-0.30	0.39
5. Going your way	2.82	0.74	0.12	-0.11	0.34
6. Could not cope	2.54	0.73	0.18	0.54	0.30
7. Control irritations	2.86	0.80	-0.01	-0.20	0.39
8. On top of things	3.16	0.92	0.02	-0.49	0.44
9. Been angered	2.48	0.86	0.46	0.31	0.36
10. Could not overcome	2.44	0.87	0.53	0.50	0.39

*Note.* PSS-10: 10-Item Perceived Stress Scale; SD: standard deviation; DI: discrimination index

### Factor structure of PSS-10

**Fig. 1:**
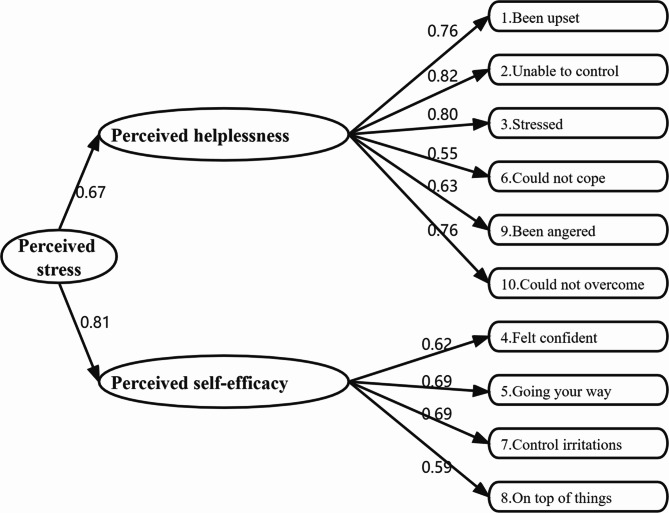
Standardized factor loadings for the two-factor structure model of the 10-item Perceived Stress Scale among Chinese nurses (N = 708)

The CFA examined the factor structure of the PSS-10. The two-factor model, comprised of negative items (Factor 1: perceived helplessness) and positive items (Factor 2: perceived self-efficacy), showed a good fit (*χ*^2^/*df =* 6.25, *p* < 0.001; CFI = 0.94, NNFI = 0.92, TLI = 0.91, RMSEA = 0.08, SRMR = 0.05). As shown in Fig. 1, the factor loadings for perceived helplessness and perceived self-efficacy were 0.67 and 0.81, respectively, and all loadings ranged from 0.55 to 0.82. In summary, CFA provided evidence for a two-factor structure model of the PSS-10.

### Reliability assessment

The Cronbach’s α for the PSS-10 total score was 0.85, and for the two subscales were 0.87 (perceived helplessness) and 0.74 (perceived self-efficacy), respectively, which indicated adequate internal consistency reliability. To confirm the test-retest reliability, 182 participants (116 females and 66 males; mean age = 31.74, SD = 7.38) completed the PSS-10 twice within a 3-month interval. The test-retest correlation coefficients were acceptable for the total score (*r* = 0.66, *p <* 0.001) and for the two subscales (perceived helplessness: *r* = 0.67, *p <* 0.001; perceived self-efficacy: *r* = 0.55, *p <* 0.001).

### Convergent validity and discriminant validity

Table 3 shows the descriptive statistics (i.e., mean, SD, Skewness and Kurtosis) and bivariate correlations of the study measures. Given that all scores of the measures were normally distributed with the absolute skewness and kurtosis values being smaller than 1 [53], Pearson correlation was used to explore the association of PSS-10 with other measures. PSS-10 score was positively correlated with neuroticism (*r* = 0.67, *p <* 0.001), and negatively correlated with extraversion (*r* = -0.38, *p <* 0.001), agreeableness (*r* = -0.40, *p <* 0.001), conscientiousness (*r* = -0.43, *p* < 0.001), and openness (*r* = -0.31, *p* < 0.001). Importantly, we compared the absolute correlation values between the PSS-10 and BFI-44 neuroticism with PSS-10 and the other BFI-44 facets. The results found that PSS-10 score was more strongly associated with neuroticism than extraversion (Steiger’s Z test: Z = 9.26, *p* < 0.001), agreeableness (Steiger’s Z test: Z = 8.48, *p* < 0.001), conscientiousness (Steiger’s Z test: Z = 7.83, *p* < 0.001), and openness (Steiger’s Z test: Z = 10.46, *p* < 0.001). These results suggest that PSS-10 was more correlated with conceptually related constructs (i.e., BFI-44 neuroticism is characterized by emotional instability, anxiety, depression, and stress) than conceptually unrelated constructs (i.e., the other BFI-44 facets). In summary, the PSS-10 showed good convergent and discriminant validity.

**Table 3:** Descriptive statistics and bivariate correlations of study measures

Measure	Mean ± SD	Skewness	Kurtosis	1	2	3	4	5	6	7	8	9	10
1. PSS-10	26.98 ± 5.58	0.23	0.69	–									
2. Perceived helplessness	15.52 ± 4.06	0.44	0.67	0.91	–								
3. Perceived self-efficacy	11.47 ± 2.51	-0.06	-0.01	0.75	0.41	–							
4. Extraversion	25.16 ± 4.19	-0.07	0.02	-0.38	-0.29	-0.38	–						
5. Agreeableness	35.34 ± 3.94	-0.12	0.09	-0.40	-0.38	-0.28	0.21	–					
6. Conscientiousness	32.92 ± 4.61	-0.04	0.31	-0.43	-0.34	-0.42	0.30	0.50	–				
7. Neuroticism	22.94 ± 4.83	0.01	-0.01	0.67	0.62	0.47	-0.45	-0.46	-0.49	–			
8. Openness	32.10 ± 4.87	0.22	0.18	-0.31	-0.19	-0.38	0.42	0.28	0.35	-0.36	–		
9. Anxiety	12.44 ± 4.01	0.84	0.70	0.64	0.65	0.37	-0.26	-0.40	-0.35	0.57	-0.16	–	
10. Depression	11.68 ± 3.76	0.82	0.69	0.68	0.66	0.45	-0.33	-0.40	-0.35	0.59	-0.21	0.83	–
11. Stress	13.87 ± 4.17	0.29	-0.17	0.71	0.74	0.40	-0.26	-0.39	-0.34	0.66	-0.20	0.83	0.83

*Note.* All correlation coefficients were statistically significant at the 0.001 level. PSS-10: 10-item Perceived Stress Scale; SD: standard deviation

### Criterion validity

To evaluate the predictive and concurrent validity of PSS-10, we first conducted correlation analyses to test the relationship between scores of the PSS-10 and the DASS-21. As shown in Table 3, PSS-10 scores were positively correlated with scores of anxiety, depression, and stress in the DASS-21. Next, we evaluated incremental validity based on hierarchical regression analyses to demonstrate whether PSS-10 could significantly predict anxiety, depression, and stress after controlling for other variables. As depicted in Table 4, the PSS-10 score explained an additional variance of 11% when predicting anxiety (*R2* = 0.11, *β* = 0.45, *p* < 0.001), an additional variance of 12% when predicting depression (*R2* = 0.12, *β* = 0.49, *p* < 0.001), and an additional variance of 13% when predicting stress (*R2* = 0.13, *β* = 0.50, *p* < 0.001) beyond the variance explained by variables in the first level. Therefore, the PSS-10 demonstrated excellent criterion validity for predicting anxiety, depression and stress.

**Table 4:** Hierarchical regression models for predicting anxiety, depression and stress

Predictor variable		Anxiety			Depression				Stress			
	B		β	R2		B	β	R2		B	β	R2
First level				0.37***				0.42***				0.48***
Sex	0.10		0.01			-0.38	-0.03			-0.36	-0.03	
Age	0.03		0.05			0.16	0.32**			0.18	0.32**	
Education level	0.07		0.01			-0.13	-0.02			0.21	0.03	
Professional title	-0.57		-0.10*			-0.60	-0.12*			-0.64	-0.11*	
Length of nursing work	0.06		0.13			-0.03	-0.06			-0.06	-0.11	
Extraversion	-0.02		-0.02			-0.06	-0.07			0.06	0.06	
Agreeableness	-0.18		-0.17***			-0.14	-0.15***			-0.12	-0.12***	
Conscientiousness	-0.07		-0.08			-0.06	-0.08*			-0.02	-0.03	
Neuroticism	0.40		0.49***			0.38	0.49***			0.56	0.65***	
Openness	0.08		0.09**			0.05	0.06			0.04	0.05	
Second level				0.48***				0.54***				0.61***
Sex	0.48		0.04			0.01	0.00			0.08	0.01	
Age	-0.00		-0.01			0.13	0.26*			0.15	0.26**	
Education level	0.12		0.02			-0.07	-0.01			0.27	0.04	
Professional title	-0.49		0.09*			-0.52	-0.10*			-0.54	-0.09*	
Length of nursing work	0.08		0.16			-0.02	-0.03			-0.04	-0.08	
Extraversion	0.02		0.02			-0.02	-0.03			0.10	0.10**	
Agreeableness	-0.13		0.13***			-0.10	-0.11**			-0.08	-0.07*	
Conscientiousness	-0.03		-0.03			-0.02	-0.03			0.02	0.02	
Neuroticism	0.20		0.24***			0.18	0.23***			0.33	0.39***	
Openness	0.09		0.11**			0.06	0.08**			0.06	0.07*	
PSS-10	0.33		0.45***			0.33	0.49***			0.37	0.50***	

*Note.* PSS-10, 10-item Perceived Stress Scale; ***p < 0.001, **p < 0.01, *p < 0.05

## Discussion

To our knowledge, this study is the first to evaluate the psychometric properties of the PSS-10 among Chinese nurses. DI suggested that all items of PSS-10 had sufficient discrimination between high-score and low-score groups. CFA showed that the PSS-10 fitted well in a two-factor structure model. Besides, the PSS-10 revealed adequate internal consistency and test-retest reliability, satisfactory convergent and discriminant validity, and criterion validity for predicting anxiety, depression and stress. Overall, our study demonstrated that the PSS-10 is suitable for measuring perceived stress levels in Chinese nurses.

The two-factor structure of PSS-10 was well supported in this study, which is consistent with previous studies based on other populations, such as some patients (e.g., asthma [60], chronic headache [57], infertility [18], mental disorders [22], and systemic lupus erythematosus [23]), normal adults [1, 17, 19, 61], and general students [20, 62]. Although the two-factor structure dominates the research field of PSS-10, a study based on 60 suicide survivors has reported a one-factor structure [63]. A possible explanation for this is that the sample size was too small for this study [13]. Additionally, the factor loadings for all items ranged from 0.55 to 0.82 in the current study, which indicated that all items of PSS-10 contributed significantly to the measurement of perceived stress in nurses. Although studies have consistently supported the two-factor structure of PSS-10, there is still a disagreement about the explanation of the two factors. For example, Roberti et al. suggested that the two dimensions could be used as subscales [64]. Nevertheless, Cohen and Williamson, the authors of the original scale, suggested that the two factors were irrelevant and only reflected item directionality [12]. Given that there are no theoretical grounds for the use of the two subscales [17], we argued that the subscale scores should be cautiously used in future studies and practices [24, 65].

Consistent with previous psychometric assessments with other languages, PSS-10 showed adequate internal consistency and test-retest reliability in this sample, which was comparable to those in other versions, such as the German version [1], the South African version [62], and the Vietnamese version [16]. In sum, PSS-10 has acceptable reliability among Chinese nurses.

Big Five personalities play an important role in the way people perceive and cope with stress [66]. Particularly, neuroticism is a personality tendency to experience negative emotions and is more likely to cope with stress in a negative way [64, 67]. Meta-analyses have shown that anxiety and depression are linked with BFI personalities, and neuroticism is the most relevant factor in this context [68]. This study extends these findings by showing that the PSS-10 score had a stronger association with neuroticism than other BFI personalities. Meanwhile, the PSS-10 score has shown a significant association with conceptually similar variables including anxiety [62] and depression [18, 25]. Moreover, the PSS-10 score could predict anxiety, depression, and stress even after controlling for other variables. Altogether, our results suggested that the PSS-10 has satisfactory convergent, discriminant and criterion validity.

This study has several limitations. First, the current sample included mostly females compared to males, thus the conclusion might be limited to female nurses. Future studies with more balanced gender are needed to examine the measurement invariance across genders [20]. Second, nursing is a special occupation, which has a complex clinical environment and a tense nurse-patient relationship. The ability to bear stress and the sensitivity to perceive stress may vary across nurses in different levels of hospitals [25, 69]. The participants in this study were from Triple-A level hospitals in southwest China, which may limit the generalizability of the findings to other nurses. Third, only self-reported measures were used in this study, thus the participants’ responses may be biased to some extent due to the impact of subjectivity like social desirability [70]. Employing more objective behavioral tests is warranted in future research to circumvent this problem.

## Conclusion

This study supported a two-factor structure of the PSS-10 among Chinese nurses, with adequate internal consistency and test-retest reliability, and satisfactory convergent, discriminant and criterion validity. In a word, PSS-10 is a reliable and valid measure of perceived stress in Chinese nurses. This scale is very short and can be filled out in a few minutes, providing a suitable measurement for future research and practice on individual stress management and coping among Chinese nurses. The findings of this study may also advance the development of psychoradiology, a burgeoning field at the intersection of psychology, psychiatry and radiology [71–74].

### Electronic supplementary material

Below is the link to the electronic supplementary material.

Supplementary Material 1

## Data Availability

The authors confirmed that all relevant data are included in the article.

## Abbreviations

BFI-44: 44-item Big Five Inventory
CFA: confirmatory factor analysis
CFI: comparative fit index
COSMIN: COnsensus-based Standards for the selection of health status Measurement INstruments
DASS-21: 21-item Depression Anxiety and Stress Scale
DI: discrimination index
NNFI: non-normal fit index
PSS: Perceived Stress Scale
PSS-10: 10-item Perceived Stress Scale
RMSEA: root mean square error of approximation
SD: standard deviations
SRMR: standardized root means square residual
TLI: Tucker-Lewis index
