# Supplementary material for: Psychometric testing of the 10-item perceived stress scale for Chinese nurses
Source: BMC Nurs. 2023 Nov 15;22:430. doi: 10.1186/s12912-023-01602-4 (PMC10647153; doi:10.1186/s12912-023-01602-4)
Supplement: Supplementary file 1 — Supplementary Material 1 [file 12912_2023_1602_MOESM1_ESM.docx]

**Supplemental Material**

**COSMIN checklist**

Box A – internal consistency

Box B – reliability

Box C – measurement error

Box D – content validity

Box E – structural validity

Box F – hypotheses testing

Box G – cross‐cultural validity

Box H – criterion validity

Box I – responsiveness

Box J – interpretability

Box generaliability

**Box A. Internal consistency**

**yes no ?**

1 Does the scale consist of effect indicators, i.e. is it based on a reflective model? ☑ ☐ ☐

*Design requirements* **yes no ?**

2 Was the percentage of missing items given?

☑ ☐

3

4

5

Was there a description of how missing items were handled?

Was the sample size included in the internal consistency analysis adequate?

Was the unidimensionality of the scale checked? i.e. was factor analysis or IRT model applied?

☑ ☐

☑

☐

☐

☑

☐

6

7

Was the sample size included in the unidimensionality analysis adequate?

Was an internal consistency statistic calculated for each (unidimensional) (sub)scale separately?

☑ ☐ ☐

☑

☐

☐

8 Were there any important flaws in the design or methods of the study?

*Statistical methods*

☐ ☑

**yes no NA**

1. for Classical Test Theory (CTT): Was Cronbach’s alpha calculated?
2. for dichotomous scores: Was Cronbach’s alpha or KR-20 calculated?
3. for IRT: Was a goodness of fit statistic at a global level calculated? e.g. χ^2^, reliability coefficient of estimated latent trait value (index of (subject or item)

separation)

☑ ☐ ☐

☐

☐

☑

☐

☐

☑

**Box B. Reliability: relative measures (including test-retest reliability, inter-rater reliability and intra-rater reliability)**

*Design requirements* **yes no ?**

1. Was the percentage of missing items given? ☑ ☐
2. Was there a description of how missing items were handled? ☑ ☐
3. Was the sample size included in the analysis adequate? ☑ ☐ ☐
4. Were at least two measurements available? ☑ ☐
5. Were the administrations independent? ☑ ☐ ☐
6. Was the time interval stated? ☑ ☐
7. Were patients stable in the interim period on the construct to be measured? ☐ ☐ ☑
8. Was the time interval appropriate? ☑ ☐ ☐
9. Were the test conditions similar for both measurements? e.g. type of administration, environment, instructions
10. Were there any important flaws in the design or methods of the study?

|  | ☐ | ☑ |  |  |  |
| --- | --- | --- | --- | --- | --- |
| **yes** | **no** | **NA** |  |  | **?** |
| ☑ | ☐ | ☐ |  |  |  |
| ☐  ☐  ☐ | ☐  ☐  ☐ | ☑  ☑  ☑ |  |  | ☐ |

*Statistical methods*

1. for continuous scores: Was an intraclass correlation coefficient (ICC) or a test-retest Pearson correlation calculated?
2. for dichotomous/nominal/ordinal scores: Was kappa calculated?
3. for ordinal scores: Was a weighted kappa calculated?
4. for ordinal scores: Was the weighting scheme described? e.g. linear, quadratic

☑ ☐ ☐

**Box C. Measurement error: absolute measures**

*Design requirements*

**yes no ?**

1 Was the percentage of missing items given?

2 Was there a description of how missing items were handled? 3 Was the sample size included in the analysis adequate?

4 Were at least two measurements available?

5 Were the administrations independent?

6 Was the time interval stated?

7 Were patients stable in the interim period on the construct to be measured? 8 Was the time interval appropriate?

9 Were the test conditions similar for both measurements? e.g. type of

administration, environment, instructions

☑ ☐

☑

☐

☑

☐

☐

☑

☐

☑

☐

☐

☑

☐

☐

☐

☑

☑

☐

☐

☑

☐

☐

10 Were there any important flaws in the design or methods of the study?

*Statistical methods*

☐ ☑

**yes no ?**

11 for CTT: Was the Standard Error of Measurement (SEM), Smallest Detectable

Change (SDC) or Limits of Agreement (LoA) calculated?

☑ ☐

**Box D. Content validity (including face validity)**

*General requirements*

**yes no ?**

1

Was there an assessment of whether all items refer to relevant aspects of the

construct to be measured?

☑ ☐ ☐

2

Was there an assessment of whether all items are relevant for the study

population? (e.g. age, gender, disease characteristics, country, setting)

☑ ☐ ☐

3

Was there an assessment of whether all items are relevant for the purpose of the

measurement instrument? (discriminative, evaluative, and/or predictive)

☑ ☐ ☐

4

Was there an assessment of whether all items together comprehensively reflect

the construct to be measured?

☑ ☐ ☐

5 Were there any important flaws in the design or methods of the study?

☐ ☑

**Box E. Structural validity**

**yes no ?**

1 Does the scale consist of effect indicators, i.e. is it based on a reflective model? ☑ ☐ ☐

*Design requirements*

**yes no ?**

2

3

4

5

Was the percentage of missing items given?

Was there a description of how missing items were handled? Was the sample size included in the analysis adequate?

Were there any important flaws in the design or methods of the study?

☑ ☐

☑

☐

☑

☐

☐

*Statistical methods*

☐ ☑

**yes no NA**

6

7

for CTT: Was exploratory or confirmatory factor analysis performed?

for IRT: Were IRT tests for determining the (uni-) dimensionality of the items performed?

☑ ☐ ☐

☐

☐

☑

| **Box F. Hypotheses testing** |  | | |
| --- | --- | --- | --- |
| *Design requirements* | **yes** | **no** | **?** |
| 1 Was the percentage of missing items given? | ☑ | ☐ |  |
| 2 Was there a description of how missing items were handled? | ☑ | ☐ |  |
| 3 Was the sample size included in the analysis adequate? | ☑ | ☐ | ☐ |
| 4 Were hypotheses regarding correlations or mean differences formulated a priori  (i.e. before data collection)? | ☑ | ☐ | ☐* |
|  | **yes** | **no** | **NA** |
| 5 Was the expected *direction* of correlations or mean differences included in the | ☑ | ☐ | ☐ |
| hypotheses? |  |  |  |
| 6 Was the expected absolute or relative *magnitude* of correlations or mean | ☐ | ☑ | ☐ |
| differences included in the hypotheses? |  |  |  |
| 7 for convergent validity: Was an adequate description provided of the comparator | ☑ | ☐ |  |
| instrument(s)? |  |  |  |
| 8 for convergent validity: Were the measurement properties of the comparator | ☑ | ☐ |  |
| instrument(s) adequately described? |  |  |  |
| 9 Were there any important flaws in the design or methods of the study? | ☐ | ☑ |  |
| *Statistical methods* | **yes** | **no** | **NA** |
| 10 Were design and statistical methods adequate for the hypotheses to be tested? | ☑ | ☐ | ☐ |
|  |  |  |  |

**Box G. Cross-cultural validity**

*Design requirements*

**yes no ?**

1

2

3

4

Was the percentage of missing items given?

Was there a description of how missing items were handled? Was the sample size included in the analysis adequate?

Were both the original language in which the HR-PRO instrument was developed,

and the language in which the HR-PRO instrument was translated described?

☑ ☐

☑

☐

☑

☐

☐

☑

☐

5

Was the expertise of the people involved in the translation process adequately described? e.g. expertise in the disease(s) involved, expertise in the construct to

be measured, expertise in both languages

☑ ☐

6

7

8

Did the translators work independently from each other?

Were items translated forward and backward?

Was there an adequate description of how differences between the original and

translated versions were resolved?

☐ ☐ ☑

☑

☐

☐

☑

☐

9

10

Was the translation reviewed by a committee (e.g. original developers)?

Was the HR-PRO instrument pre-tested (e.g. cognitive interviews) to check interpretation, cultural relevance of the translation, and ease of comprehension?

☑ ☐

☑

☐

11

12

Was the sample used in the pre-test adequately described?

Were the samples similar for all characteristics except language and/or cultural background?

☑ ☐

☑

☐

☐

13 Were there any important flaws in the design or methods of the study?

*Statistical methods*

☐ ☑

**yes no NA**

1. for CTT: Was confirmatory factor analysis performed?
2. for IRT: Was differential item function (DIF) between language groups assessed?

☑

☐ ☐

☐

☐

☑

**Box H. Criterion validity**

*Design requirements*

**yes no ?**

1

2

3

4

Was the percentage of missing items given?

Was there a description of how missing items were handled? Was the sample size included in the analysis adequate?

Can the criterion used or employed be considered as a reasonable ‘gold

standard’?

☑ ☐

☑

☐

☑

☐

☐

☑

☐

☐

5 Were there any important flaws in the design or methods of the study?

*Statistical methods*

☐ ☑

**yes no NA**

6

for continuous scores: Were correlations, or the area under the receiver operating

curve calculated?

☑ ☐ ☐

7 for dichotomous scores: Were sensitivity and specificity determined?

☐ ☐ ☑

**Box I. Responsiveness**

*Design requirements* **yes no ?**

| 1 | Was the percentage of missing items given? | ☑ | ☐ |  |
| --- | --- | --- | --- | --- |
| 2 | Was there a description of how missing items were handled? | ☑ | ☐ |  |
| 3 | Was the sample size included in the analysis adequate? | ☑ | ☐ | ☐ |
| 4 | Was a longitudinal design with at least two measurement used? | ☑ | ☐ |  |
| 5 | Was the time interval stated? | ☑ | ☐ |  |
| 6 | If anything occurred in the interim period (e.g. intervention, other relevant events), | ☐ | ☑ |  |
|  | was it adequately described? |  |  |  |
| 7 | Was a proportion of the patients changed (i.e. improvement or deterioration)? | ☐ | ☑ | |
| ***Design requirements for hypotheses testing*** | | **yes** | **no** | **?** |
| For constructs for which a gold standard was not available: | |  |  |  |
| 8 Were hypotheses about changes in scores formulated a priori (i.e. before data collection)? | | ☑ | ☐ | ☐* |
|  | | **yes** | **no** | **NA** |
| 9 Was the expected *direction* of correlations or mean differences of the change | | ☑ | ☐ | ☐ |
| scores of HR-PRO instruments included in these hypotheses? | |  |  |  |
| 10 Were the expected absolute or relative *magnitude* of correlations or mean | | ☐ | ☑ | ☐ |
| differences of the change scores of HR-PRO instruments included in these | |  |  |  |
| hypotheses? | |  |  |  |
| 11 Was an adequate description provided of the comparator instrument(s)? | | ☑ | ☐ |  |
| 12 Were the measurement properties of the comparator instrument(s) adequately | | ☑ | ☐ |  |
| described? | |  |  |  |
| 13 Were there any important flaws in the design or methods of the study? | | ☐ | ☑ |  |
| *Statistical methods* | | **yes** | **no** | **NA** |
| 14 Were design and statistical methods adequate for the hypotheses to be tested? | | ☑ | ☐ | ☐ |
| ***Design requirement for comparison to a gold standard*** | | **yes** | **no** | **?** |
| For constructs for which a gold standard was available: | |  |  |  |
| 15 Can the criterion for change be considered as a reasonable gold standard? | | ☑ | ☐ | ☐ |
| 16 Were there any important flaws in the design or methods of the study? | | ☐ | ☑ |  |

*Statistical methods*

**yes no NA**

17 for continuous scores: Were correlations between change scores, or the area

under the Receiver Operator Curve (ROC) curve calculated?

☑ ☐ ☐

18 for dichotomous scales: Were sensitivity and specificity (changed versus not

changed) determined?

☐ ☐ ☑

| **Box J. Interpretability** | **yes** | **no** | **?** |
| --- | --- | --- | --- |
| 1 Was the percentage of missing items given? | ☑ | ☐ |  |
| 2 Was there a description of how missing items were handled? | ☑ | ☐ |  |
| 3 Was the sample size included in the analysis adequate? | ☑ | ☐ | ☐ |
| 4 Was the distribution of the (total) scores in the study sample described? | ☑ | ☐ |  |
| 5 Was the percentage of the respondents who had the lowest possible (total) score described? | ☐ | ☑ |  |
| 6 Was the percentage of the respondents who had the highest possible (total) score described? | ☐ | ☑ |  |
| 7 Were scores and change scores (i.e. means and SD) presented for relevant (sub) groups? e.g. for normative groups, subgroups of patients, or the general population | ☐ | ☑ |  |
| 8 Was the minimal important change (MIC) or the minimal important difference (MID) determined? | ☐ | ☑ |  |
| 9 Were there any important flaws in the design or methods of the study? | ☐ | ☑ |  |

**Box Generalisability box**

**yes no NA**

Was the sample in which the HR-PRO instrument was evaluated adequately

described? In terms of:

1

2

3

median or mean age (with standard deviation or range)? distribution of sex?

important disease characteristics (e.g. severity, status, duration) and

description of treatment?

☑ ☐

☑

☐

☐

☐

☑

4

setting(s) in which the study was conducted? e.g. general population,

primary care or hospital/rehabilitation care

☑ ☐

5

6

countries in which the study was conducted?

language in which the HR-PRO instrument was evaluated?

☑ ☐

☑

☐

7

Was the method used to select patients adequately described? e.g. convenience,

consecutive, or random

☑ ☐

**yes no ?**

8 Was the percentage of missing responses (response rate) acceptable? ☑ ☐ ☐
